# A nomogram for the prediction of the survival of patients with advanced non‐small cell lung cancer and interstitial lung disease

**DOI:** 10.1002/cam4.5852

**Published:** 2023-03-31

**Authors:** Weigang Xiu, Jincheng Zheng, Yuwen Zhou, He Du, Jiayu Li, Wei Li, Fei Zhou, Caicun Zhou, Fengying Wu

**Affiliations:** ^1^ Department of Thoracic Oncology and State Key Laboratory of Biotherapy, Cancer Center, West China Hospital Sichuan University Chengdu 610041 PR China; ^2^ Jinhua Municipal Central Hospital, Affiliated Jinhua Hospital Zhejiang University School of Medicine Zhejiang 321099 PR China; ^3^ Department of Medical Oncology, Shanghai Pulmonary Hospital Tongji University School of Medicine Shanghai 200433 PR China

**Keywords:** advanced non‐small cell lung cancer, chemotherapy, interstitial lung disease, nomogram, predictive factor

## Abstract

**Background:**

Lung cancer is frequently accompanied by interstitial lung disease (ILD), and the overall survival (OS) of patients with these comorbidities is poor. Thus, we developed a nomogram for the prediction of the OS of patients with advanced non‐small cell lung cancer (NSCLC) and ILD.

**Patients and Methods:**

Patients with wild‐type gene advanced NSCLC with and without ILD who underwent chemotherapy between 2014 and 2019 were enrolled in the present study. The 0.5‐ and 1‐year progression‐free survival (PFS) and overall survival (OS) times of patients with and without ILD were determined using the Kaplan–Meier method. Cox regression was used to assess the prognostic value of clinical factors for patients with ILD. Based on the multivariate regression results, a nomogram for survival prediction was developed. The nomogram was validated using calibration curve.

**Results:**

Data from 155 patients with lung cancer and ILD and 118 matched patients with lung cancer alone who were receiving first‐line chemotherapy were analyzed. The first‐line chemotherapy regimens were paclitaxel + carboplatin, pemetrexed + carboplatin, gemcitabine + carboplatin, and other. The median PFS and OS were significantly shorter in patients with than in those without ILD (3.0 vs. 7.0 months [*p* < 0.001] and 7.0 vs. 15.0 months (*p* < 0.001), respectively). Multivariate analysis showed that the lymphocyte count (hazard ratio [HR] 2.38; 95% confidence interval [CI], 1.44–3.94; *p* = 0.01), partial pressure of oxygen (PaO_2_; HR, 1.37; 95% CI, 1.03–1.82; *p* = 0.03), and chemotherapy regimen were independently associated with prognosis. The nomogram showed good discriminatory ability [C‐index = 0.69 (95% CI, 0.49–0.82)]. Calibration curves showed that predicted and actual prognoses were consistent.

**Conclusion:**

This nomogram can aid the prediction of the OS of patients with advanced NSCLC and ILD.

## Graphical abstract

A nomogram for the prediction of the survival of patients with advanced non‐small cell lung cancer and intersitial lung disease.

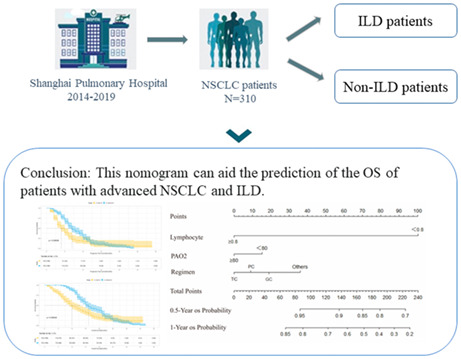

## 1 INTRODUCTION

Lung cancer is among the most common malignancies, causing about 25% of all cancer‐related deaths worldwide.^1^ Nearly 85% of lung cancers diagnosed are non‐small‐cell lung cancer (NSCLC),^2^ for which interstitial lung disease (ILD) is an independent risk factor.^3^ The rate of NSCLC/ILD comorbidity is high (5.8%–15.2%).^4^

NSCLC and ILD have common underlying pathogenic mechanisms.^5^ Epithelial dysfunction, abnormal growth factor secretion, and the weakening of cellular interaction have been reported to be key factors in the cancerous transformation of epithelial cells.^6^, ^7^ Although extensive epidemiological and mechanistic links between ILD and lung cancer have been identified, little evidence that could guide the assessment and treatment of these co‐occurring diseases is available. An optimal treatment for NSCLC with ILD and the factors with potential prognostic impact need to be identified.^8^ Patients with ILD have been excluded from most clinical trials of lung cancer, especially those examining NSCLC.^9^ Advanced NSCLC can be treated with targeted molecular therapy, chemotherapy, and immune‐checkpoint inhibitors (ICIs),^10^ but the administration of tyrosine kinase inhibitors and ICIs tends to be avoided in patients with comorbid ILD due to the risk of acute exacerbation (AE‐ILD).^9^, ^10^ For patients with advanced NSCLC and ILD who have undergone first‐line cytotoxic chemotherapy, the median progression‐free survival (PFS) and overall survival (OS) times are 5.3 and 10.6 months, respectively, worse than those for patients with advanced NSCLC and no comorbidity.^11^ In the present study, we attempted to build and internally validate a nomogram for the prediction of OS for patients with NSCLC and ILD receiving first‐line chemotherapy.

## 2 MATERIALS AND METHODS

### 2.1 Patients and data collection

We retrospectively analyzed data from 310 patients with advanced NSCLC with and without comorbid ILD who received first‐line chemotherapy at Shanghai Pulmonary Hospital between January 2014 and December 2019. TNM stages were defined according to the 8th edition of the American Joint Committee on Cancer/Union for International Cancer Control guidelines.^12^ The inclusion criteria were: (1) histologically confirmed stage IIIB, IIIC, or IV NSCLC; (2) first‐line chemotherapy as the sole therapy; (3) Eastern Cooperative Oncology Group (ECOG) performance status of 0–2; and (4) no epidermal growth factor receptor, anaplastic lymphoma kinase, or ROS1 alteration. The exclusion criteria were: (1) pneumoconiosis, autoimmune disease, or other known secondary ILD; and (2) loss to follow‐up or incomplete medical data. The patients received no other treatment (e.g., palliative radiotherapy, immunotherapy, or targeted therapy). The potential predictive factors were the lymphocyte count, pulmonary partial oxygen pressure (PaO_2_), and serum protein electrophoresis. The cut‐off values used for these factors were based on the literature and the hospital laboratory's parameters.^13^ They were measured at the time of lung cancer diagnosis and confirmation by pathological examination. The following data were collected: age, sex, smoking history, histological findings, clinical stage, ECOG performance status, lesion location, lymphocyte count, chemotherapy regimen, pulmonary function, PaO_2_, and findings of serum protein electrophoresis. The baseline characteristics (lymphocyte count, chemotherapy regimen, blood gas findings, and pulmonary function) of patients in the two groups were not balanced prior to matching (all *p* < 0.05). Demographic and clinical variables included in the propensity score model were age, sex, histological type, ECOG status, clinical stage, location, lymphocyte count, chemotherapy regimen (pemetrexed + carboplatin [PC], paclitaxel + carboplatin [TC]; gemcitabine + carboplatin [GC]; others [docetaxel, vinorelbine and S‐1]), blood gas findings, and pulmonary function. In total, 236 patients matched according to propensity scores were allocated to two groups of 118 patients each. PFS (from first‐line chemotherapy initiation to the confirmation of progression or death) and OS (from diagnosis to death of any cause or last visit) were determined. OS was recorded during follow‐up clinical visits or by telephone. All patients were followed until death or December 2019.

### 2.2 Diagnostic criteria for ILD and UIP

Diffuse parenchymal lung diseases, often referred to collectively as ILD, form a heterogeneous group of disorders that are classified together because of their similar clinical, radiographic, physiological, and/or pathological manifestations. ILD was classified according to computed tomographic patterns as usual interstitial pneumonia (UIP) and non‐UIP.^14^, ^15^ The diagnosis of the UIP pattern was based on the American Thoracic Society/European Respiratory Society/Japanese Respiratory Society/Latin American Thoracic Society clinical practice guidelines.^14^ The UIP pattern was defined as subpleural and basally predominant, often with a heterogeneous distribution, and featuring honeycombing with or without peripheral traction bronchiectasis or bronchiolectasis^14^; cases lacking these features were considered to have the non‐UIP pattern.^16^ The UIP pattern category did not include cases of probable or indeterminate UIP.

Chest HRCT (Siemens) was performed with a slice thickness of 1–2 mm. Two thoracic radiologists reviewed the images to diagnose ILD and classify cases as UIP and non‐UIP.^14^, ^17^ The severity of ILD was rated using a four‐grade system according to the percentage of fibrosis.

### 2.3 Definition of AE‐ILD

AE‐ILD is generally characterized by suddenly progressive and severe respiratory failure, with new lung opacities and diffuse alveolar damage.^18^ Exacerbation in patients without ILD was characterized by acute worsening or the development of dyspnea of another etiology.

### 2.4 Statistical analysis

The data were analyzed using SPSS 22.0 (IBM Corporation) and the R software (version 3.4.3; R Studio). Propensity scores were calculated using logistic regression and the following covariates: age, sex, smoking history, ECOG status, histology, clinical stage, lesion location, lymphocyte count, chemotherapy regimen, PaO_2_, percent forced vital capacity, percent forced expiratory volume in 1 s, and albumin level. Kaplan–Meier analysis and the log‐rank test were used to assess the PFS and OS times of patients with and without ILD. Cox proportional‐hazards regression models were used to evaluate age, gender, smoking history, ECOG status, histology, clinical stage, lesion location, lymphocyte count, chemotherapy regimen, ILD pattern, PaO_2_, and albumin level as predictors of the OS of patients with NSCLC and ILD, with the calculation of hazard ratios (HRs) and 95% confidence intervals (CIs). Multivariate analyses were performed to identify independent prognostic factors. Two‐tailed *p* values <0.05 were taken to be significant.

The independent risk factors identified were included in a nomogram developed for the prediction of the probability of post‐chemotherapy 0.5‐ and 1‐year OS among patients with advanced NSCLC and ILD. Receiver operating characteristic (ROC) and calibration curves were used to assess the nomogram's performance and discrimination ability, respectively.

## 3 RESULTS

### 3.1 Patient characteristics

Of the 310 patients with advanced NSCLC enrolled in this study (266 [85.8%] men, 44 women [14.2%]; mean age 61.9 [range, 45–82] years), 155 patients also had ILD. All patients with ILD had undergone HRCT examination. The patients were assigned randomly to development (70%[*n* = 108]) and internal validation (30% [*n* = 47]) populations. Their clinicopathological characteristics are summarized in Table 1. Most (*n* = 296 [95.5%]) patients had ECOG performance status scores of 0 or 1. NSCLC diagnoses were adenocarcinoma (*n* = 116 [37.4%]), squamous cell carcinoma (*n* = 94 [30.4%]), and other (*n* = 100 [32.2%]). Most lesions were peripheral, primarily in the lower pulmonary lobes. The longest follow‐up period was 72 months, and the median follow‐up period was 39 months (range, 6–72 months). At the end of the follow‐up period, 12 patients had been lost and 247 patients had died. Chemotherapy regimens were paclitaxel + carboplatin (*n* = 43 [13.8%]), pemetrexed + carboplatin (*n* = 56 [18.1%]), gemcitabine + carboplatin (*n* = 88 [28.4%]), and other (*n* = 123 [39.7%]). Other regimens employed single agents and were used for patients of advanced age (≥70 years) who could not tolerate platinum‐based double‐agent chemotherapy. These regimens included docetaxel (*n* = 33 [10.7%]), vinorelbine (*n* = 34 [11.0%]), and S‐1 (*n* = 56 [18.1%]). Bevacizumab was not administered to patients with lung adenocarcinoma due to pre‐existing bleeding, thrombi, and/or non‐coverage by health insurance.

**TABLE 1:** Clinical characteristics of patients with NSCLC with and without interstitial lung disease.

The original unmatched cohort				The propensity‐matched cohort
Characteristics	LC with ILD (*n* = 155)	LC without ILD (*n* = 155)	*p* value	*p* value
Age, median (range), years	66 (35–81)	62.2 (32–80)	0.17	0.37
Gender, male/female	133/22	133/22	1.00	0.97
Smoking history, yes/no	90/65	74/81	0.07	0.08
Histology, ad/sq/other NSCLC	58/45/52	58/49/48	0.63	0.65
Clinical stages, IIIB‐IIIC/IV	62/93	60/95	0.82	0.75
ECOG, 0–1/2	149/6	147/8	0.58	0.66
Location, central/peripheral	72/83	64/91	0.36	0.29
Lymphocyte, <0.8/≥0.8 × 10^9^/L	53/102	1/154	0.01	0.78
Chemotherapy regimens				
TC/PC/GC/Others	13/24/31/87	30/32/57/36	0.01	0.67
PaO_2_, ≤80/>80 mmHg	94/61	51/104	0.01	0.07
PaCO_2_, ≤45/>45 mmHg	153/2	150/5	0.05	0.41
Albumin, ≤55.8%/>55.8%	98/57	46/109	0.01	0.87
α1‐globulin, ≤2.9%/>2.9%	134/21	6/149	0.01	0.06
%FVC, median (range)	92.0 (50.2–122.2)	81.7 (40.4–119)	0.03	0.13
FEV_1_% (range)	81.5 (47.3–124.4)	74.3 (36.1–126)	0.01	0.89
%DLCO (range)	62.9 (56.7–98.9)	80.1 (29.4–109.3)	0.01	0.97

Abbreviations: %DLCO, percent diffusing capacity of the lung for carbon monoxide; %FVC, percent forced vital capacity; ad, adenocarcinoma; ECOG, Eastern Cooperative Oncology Group; FEV_1_%, percent forced expiratory volume in 1 s; GC, gemcitabine + carboplatin; LC, lung cancer; ILD, interstitial lung disease; NSCLC, non‐small cell lung cancer; PaCO_2_, partial pressure of carbon dioxide; PaO_2_, partial pressure of oxygen; PC, pemetrexed + carboplatin; sq, squamous cell carcinoma; TC, paclitaxel + carboplatin.

The mean PaO_2_ at rest was 80.5 ± 10.7 mm Hg. The mean percent predicted forced vital capacity was 92.0% (range, 50.2%–122.2%), the mean percent forced expiratory volume in 1 s was 81.5% (range, 47.3%–124.4%), and the mean diffusing capacity of the lungs for carbon monoxide was 62.9% (range, 56.7%–98.9%).

### 3.2 Survival and treatment safety

**FIGURE 1:**
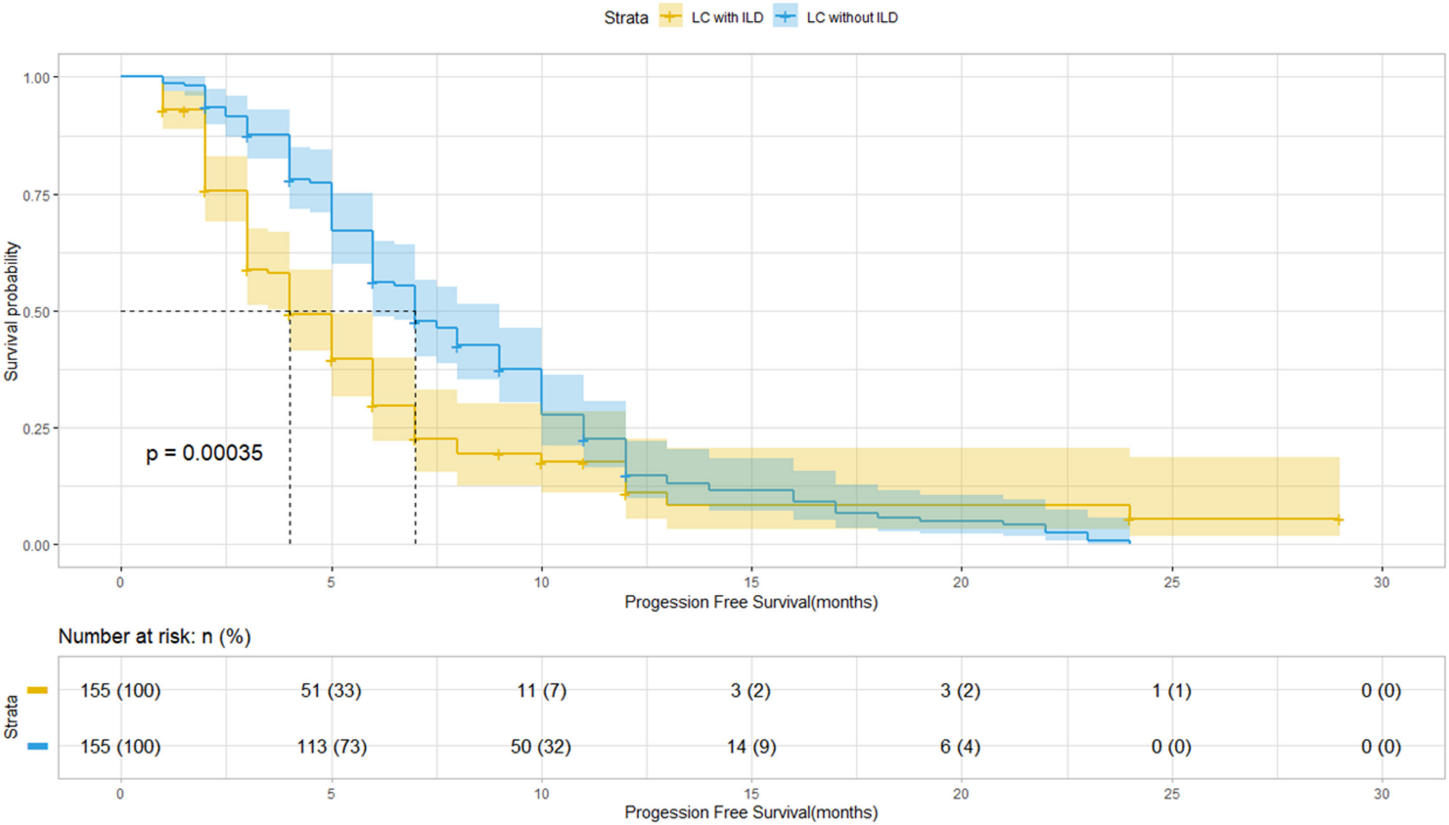
Progression‐free survival of patients with advanced non‐small cell lung cancer (LC) with and without interstitial lung disease (ILD) after first‐line chemotherapy.

**FIGURE 2:**
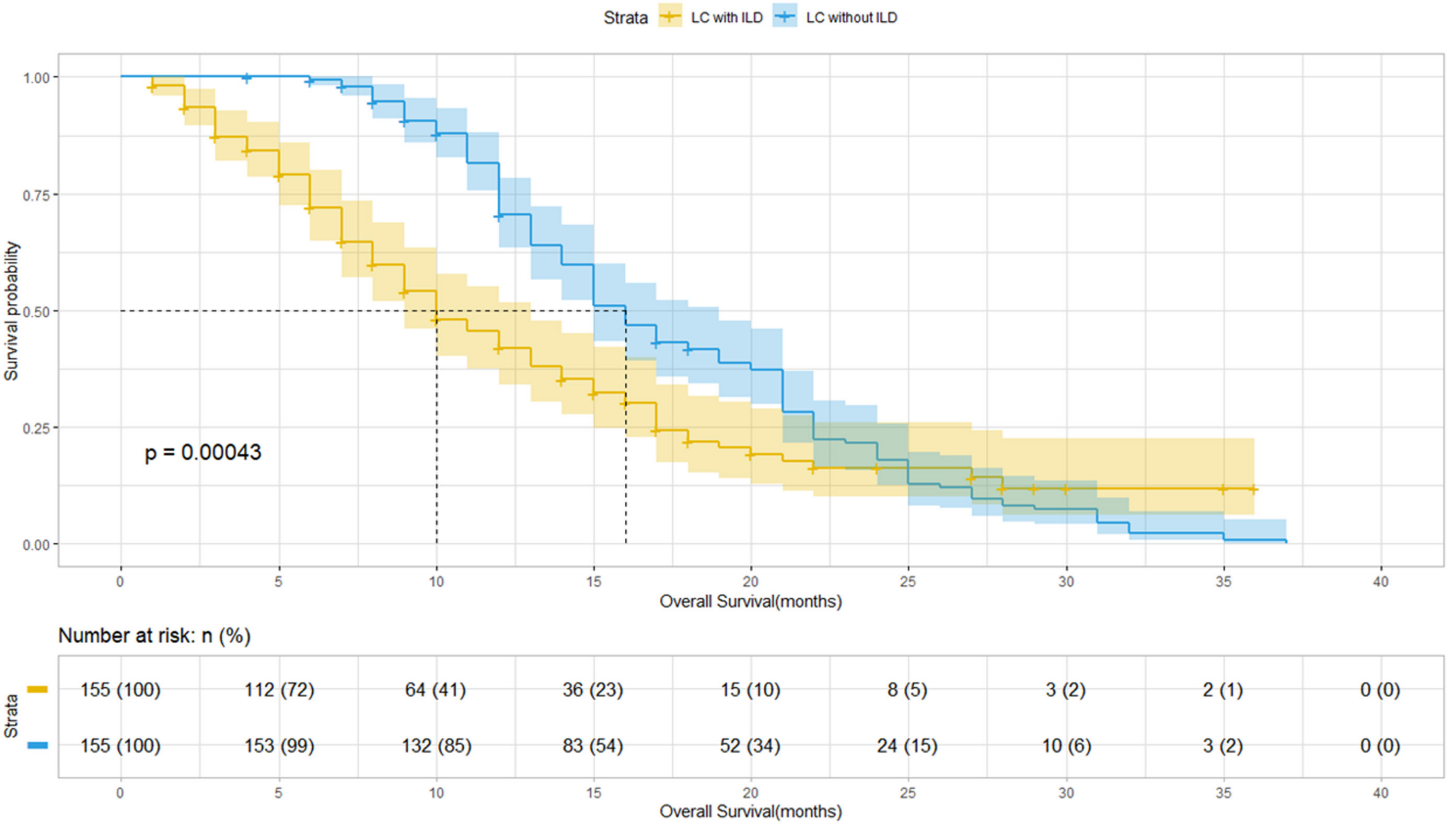
Overall survival of patients with advanced non‐small cell lung cancer (LC) with and without interstitial lung disease (ILD) after first‐line chemotherapy.

The median PFS and OS were significantly shorter in patients with than in those without ILD (3 [95% CI, 2–4] vs. 7 [95% CI, 4–10] months [*p* < 0.001] and 7 [95% CI, 5–9] vs. 15 [95% CI, 7–23] months [*p* < 0.001], respectively; Figures 1 and 2).

The overall AE‐ILD incidence rate was 12% (Table 2). In the UIP group, this rate was 14% for patients receiving paclitaxel + carboplatin, 25% for those receiving pemetrexed + carboplatin, 38% for those receiving gemcitabine + carboplatin, 36% for those receiving docetaxel, 9% for those receiving vinorelbine, and 27% for those receiving S‐1.

**TABLE 2:** Acute exacerbation of ILD.

	LC with UIP		LC with non‐UIP	
	No. of patients administered	Exacerbation of ILD (%)	No. of patients administered	Exacerbation of ILD (%)
TC	7	1 (14%)	15	1 (7%)
PC	12	3 (25%)	16	10 (6%)
GC	16	6 (38%)	29	2 (7%)
Others				
Docetaxel	11	4 (36%)	6	1 (17%)
Vinorelbine	11	1 (9%)	4	1 (25%)
S‐1	22	6 (27%)	6	1 (17%)

Abbreviations: GC, gemcitabine + carboplatin; ILD, interstitial lung disease; LC, lung cancer; PC, pemetrexed + carboplatin; TC, paclitaxel + carboplatin; UIP, usual interstitial pneumonia.

### 3.3 Results of univariate and multivariate analyses

The univariate analysis revealed significant associations of the lymphocyte count, chemotherapy regimen, and PaO_2_ with the OS of patients with NSCLC (all *p* < 0.05; Table 3). The multivariate analysis revealed that the lymphocyte number (HR, 2.38; 95% CI, 1.44–3.94; *p* = 0.01), chemotherapy regimen (GC vs. TC: HR, 1.26; 95% CI, 1.04–1.44, *p* = 0.01; GC vs. PC: HR, 1.12; 95% CI, 1.03–1.34; *p* = 0.01; GC vs. others: HR, 1.16; 95% CI, 1.02–1.24; *p* = 0.02), and PaO_2_ (HR, 1.37; 95% CI, 1.03–1.82; *p* = 0.03) were independent prognostic factors for OS.

**TABLE 3:** Overall survival of patients with NSCLC and ILD (*n* = 155).

Variable	Univariate analysis		Multivariate analysis	
	HR (95% CI)	*p* value	HR (95% CI)	*p* value
Age		0.49		0.41
≤60 versus >60 years	1.10 (0.85–1.43)		1.13 (0.84–1.52)	
Gender		0.27		0.34
Females versus males	0.80 (0.54–1.18)		0.80 (0.50–1.27)	
Smoking history		0.68		0.17
No versus Yes	1.06 (0.82–1.36)		1.24 (0.91–1.70)	
Performance status		0.44		0.29
ECOG 0–1 versus 2	1.25 (0.71–2.19)		1.39 (0.76–2.54)	
Histology		0.57		0.54
Ad versus sq versus others NSCLC	1.03 (0.92–1.15)		0.96 (0.85–1.09)	
Clinical stages		0.19		0.12
IIIB versus IIIC versus IV	1.20 (0.92–1.56)		1.27 (0.94–1.72)	
Location		0.65		0.43
Central versus peripheral	0.94 (0.73–1.22)		0.90 (0.68–1.18)	
Lymphocyte number		0.02		0.01
≤0.8 versus >0.8 × 10^9^/L	1.23 (0.84–1.79)		2.38 (1.44–3.94)	
Chemotherapy regimens				
GC versus TC	1.19 (1.07–1.24)	0.03	1.26 (1.04–1.44)	0.01
GC versus PC	1.22 (1.01–1.31)	0.02	1.12 (1.03–1.34)	0.01
GC versus Others	1.11 (1.05–1.22)	0.04	1.16 (1.02–1.24)	0.02
ILD pattern		0.33		0.66
UIP versus non‐UIP	1.22 (1.05–1.39)		1.37 (1.04–1.24)	
PaO_2_		0.03		0.03
≤80 versus >80 mmHg	1.33 (1.03–1.72)		1.37 (1.03–1.82)	
Albumin		0.05		0.05
≤55.8% versus >55.8%	1.30 (1.00–1.69)		1.39 (1.00–1.92)	
α1‐globulin		0.04		0.72
≤2.9% versus >2.9%	1.47 (1.13–1.90)		0.92 (0.57–1.47)	
%FVC	0.99 (0.83–1.18)	0.87	1.69 (0.70–4.09)	0.25
FEV_1_%	0.90 (0.77–1.05)	0.18	0.80 (0.62–1.03)	0.09
%DLCO	0.95 (0.81–1.12)	0.56	1.04 (0.85–1.26)	0.73

Abbreviations: %DLCO, percent diffusing capacity of the lung for carbon monoxide; %FVC, percent forced vital capacity; ad, adenocarcinoma; CI, confidence interval; ECOG, Eastern Cooperative Oncology Group; FEV_1_%, percent forced expiratory volume in 1 s; GC, gemcitabine + carboplatin; HR, hazard ratio; ILD, interstitial lung disease; NSCLC, non‐small cell lung cancer; PaO_2_, partial pressure of oxygen; PC, pemetrexed + carboplatin; sq, squamous cell carcinoma; TC, paclitaxel + carboplatin; UIP, usual interstitial pneumonia.

### 3.4 Nomogram development and validation

**FIGURE 3:**
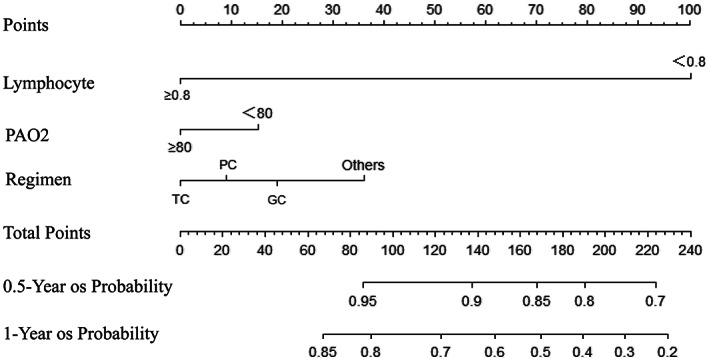
Nomogram for the prediction of 0.5‐ and 1‐year overall survival of patients with non‐small cell lung cancer. PaO_2_, partial pressure of oxygen; TC, paclitaxel + carboplatin; PC, pemetrexed + carboplatin; GC, gemcitabine + carboplatin.

**FIGURE 4:**
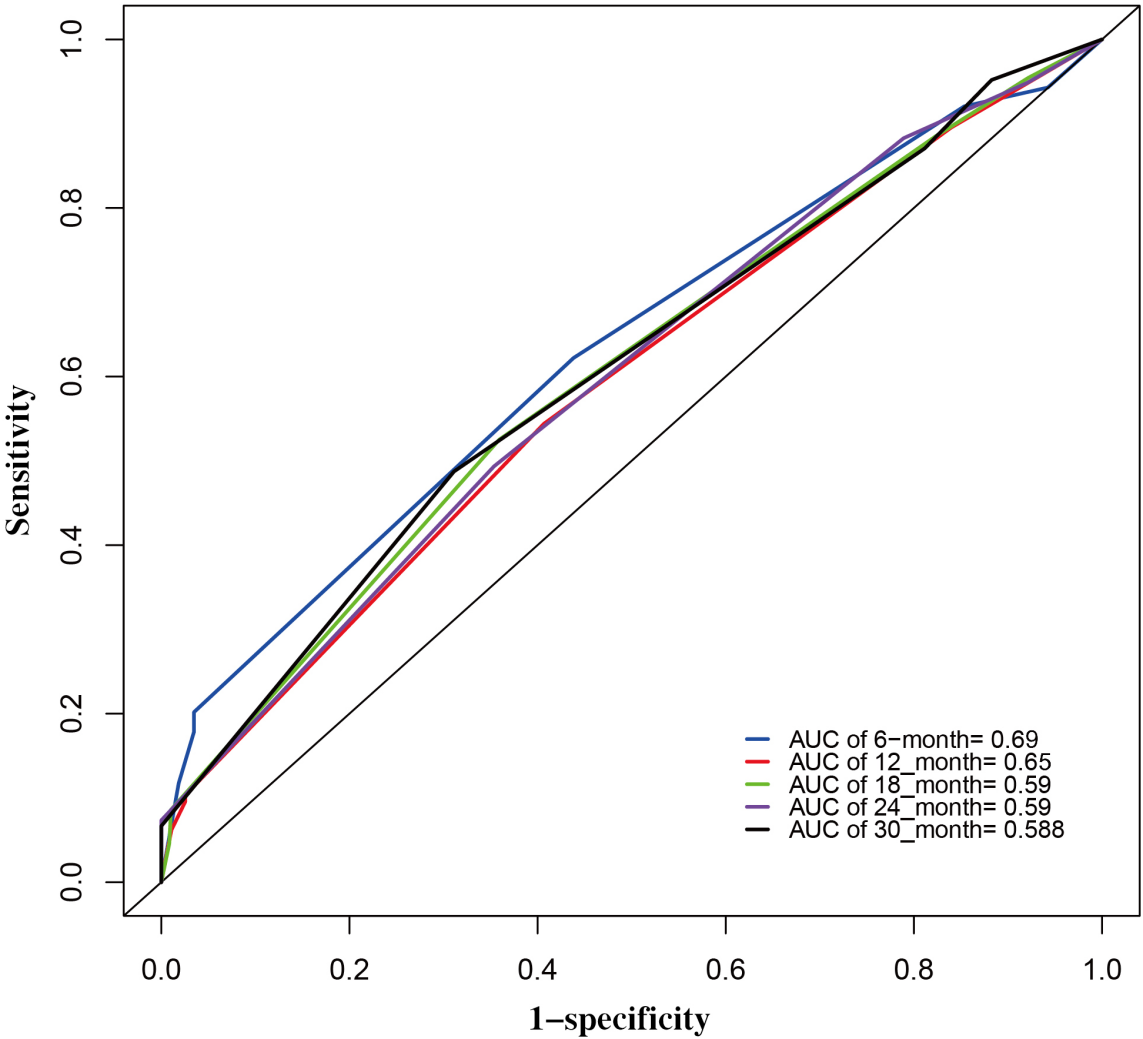
Receiver operating characteristic curves for 0.5‐ and 1‐year survival in the training cohort. AUC, area under the curve.

**FIGURE 5:**
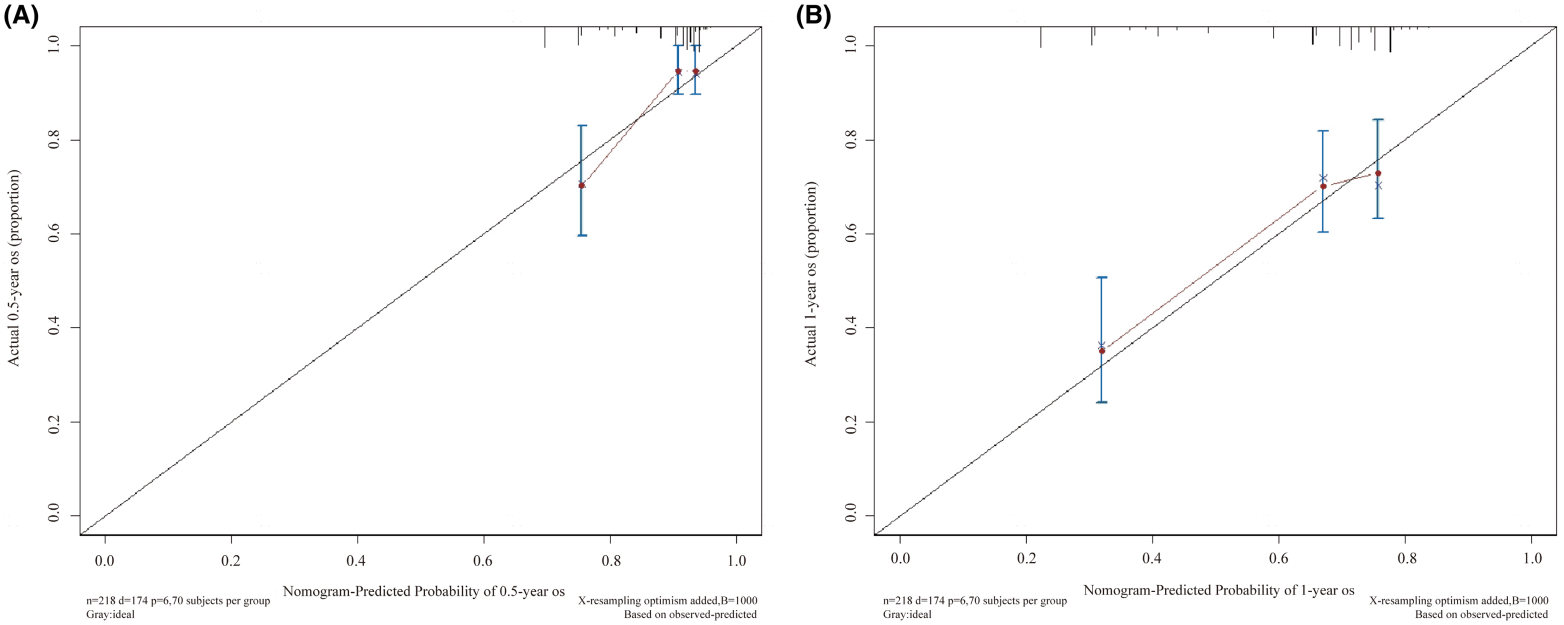
Calibration curves for the prediction of 0.5‐year (A) and 1‐year (B) overall survival (OS) in the training cohort.

The nomogram for OS prediction was based on the independent risk factors of the lymphocyte count (≤0.8 or >0.8 × 10^9^/L), chemotherapy regimen (paclitaxel + carboplatin, pemetrexed + carboplatin, gemcitabine + carboplatin, or other), and PaO_2_ (≤80 or >80 mm Hg; Figure 3). Total scores (predictions of 0.5‐ and 1‐year OS) were calculated by summing the individual factor scores. ROC curves and the C‐index (0.69 [95% CI, 0.49–0.82]) showed that the nomogram had good discriminatory ability (Figure 4). The calibration curves demonstrated consistency of actual and predicted 0.5‐ and 1‐year OS in the development and validation cohorts (Figure 5).

## 4 DISCUSSION

In this study, we determined that the lymphocyte count, chemotherapy regimen, and PaO_2_ affected the clinical outcomes of patients with NSCLC and ILD after first‐line chemotherapy and developed a clinical nomogram for prognosis prediction based on these factors. Our model demonstrated that patients with ILD; lymphocyte counts ≤0.8 × 10^9^/L; docetaxel, vinorelbine, and S‐1 chemotherapy regimens; and PaO_2_s ≤80 mmHg have worse OS. We also showed that patients with NSCLC and ILD have significantly shorter PFS and OS times than do those with NSCLC without ILD, similar to previous reports.^15^, ^19^, ^20^ Alomaish et al.^15^ found that the 5‐year survival rates for patients with lung cancer with and without ILD were 41% and 48%, respectively, and that ILD comorbidity increased the risk of death by 1.522 times. Kenmotsu et al.^19^ reported median post–platinum‐based chemotherapy PFS and OS times of 4.8 and 9.9 months, respectively, for patients with lung cancer and ILD; this OS was poorer than that for patients with lung cancer without ILD (11–15 months).

As few prospective studies have involved the assessment of chemotherapy regimens for patients with advanced NSCLC and ILD,^11^, ^21^, ^22^ the optimal regimen has not been identified.^23^ AE‐ILD, among most common causes of death for these patients, must be avoided.^24^ The mechanism of AE‐ILD may involve the elevation of levels of factors such as Krebs von den Lungen 6, surfactant protein D, and lysyl oxidase.^18^, ^25^, ^26^ Chemotherapy‐related AE‐ILD has diverse clinical manifestations, ranging from the absence of symptoms to fatal respiratory failure^27^; reported incidence and mortality rates for this condition are 5.6–43% and 27.9%, respectively.^11^, ^28^, ^29^ Minegishi et al.^11^ reported median PFS and OS times of 5.3 and 10.6 months, respectively, after the receipt of carboplatin plus weekly paclitaxel for patients with inoperable NSCLC and ILD. Notably, 5.6% of patients with ILD experienced AE‐ILD. Thus, the selection of chemotherapy regimens for patients with NSCLC and ILD in clinical practice must be performed with caution. Docetaxel and gemcitabine have been associated with drug‐induced ILD and AE‐ILD.^30^, ^31^ In contrast, Kenmotsu and colleagues^32^ found that vinorelbin and paclitaxel were rarely responsible for AE‐ILD (incidence rates of 0% and 3%, respectively, vs. 28% for docetaxel and 42% for gemcitabine). In our study, paclitaxel + carboplatin was associated with a lower AE‐ILD rate and better median OS, whereas the opposite was observed for gemcitabine + carboplatin and docetaxel, suggesting that the latter regimens should be avoided for patients with NSCLC and ILD.

In our study, peripheral blood lymphocyte counts <0.8 × 10^9^/L implied worse survival for patients with NSCLC and ILD; this count is an indicator of immunological status, which can be affected by chemotherapy in patients with NSCLC.^33^ Aldarouish et al.^34^ observed a reduced mean CD8+ T‐cell count and increased mean T helper 1 cell count in the peripheral blood of patients with NSCLC who had undergone chemotherapy relative to counts obtained from healthy subjects. Similarly, Conesa et al.^35^ found a reduced peripheral blood lymphocyte count (<1500 × 10^6^/L) after primary chemotherapy in patients with breast cancer, which was associated with reduced disease‐free survival.

PaO_2_ reduction is a frequently reported independent predictor of unfavorable postoperative prognoses, including AE‐ILD, in patients with lung cancer.^36^ A recent meta‐analysis^37^ of 15 studies demonstrated that a reduced ratio of PaO_2_ to the fraction of inspired oxygen was associated with all‐cause mortality in patients with AE‐ILD. In our study, PaO_2_ <80 mm Hg tended to associated with shorter survival times in patients with advanced NSCLC and ILD relative to those without ILD. Mechanisms potentially underlying this association include the development of chemotherapy drug resistance or an aggressive phenotype, which worsen prognoses, due to tumor hypoxia.^38^

Several limitations of this study should be considered. First, as it was retrospective, selection bias cannot be ruled out fully. Second, all data were collected from a single institution. However, the treatments administered to patients in our sample align with the current National Comprehensive Cancer Network guidelines^39^ and expert consensus^9^; thus, the study findings may aid decision making regarding the treatment of patients with advanced NSCLC. Third, the sample was small, limiting the generalizability of the findings. Fourth, although the nomogram developed in this study was validated internally, external validation is needed. Finally, although the nomogram is based on readily available clinical data, facilitating survival prediction for patients with advanced NSCLC, the inclusion of additional clinical variables would be beneficial.

In conclusion, the lymphocyte count, PaO_2_, and chemotherapy regimen were identified as independent risk factors for patients with NSCLC and ILD who have undergone first‐line chemotherapy in this study. The nomogram developed and internally validated in this study could be useful for survival prediction for this population in clinical practice. Although further validation of the nomogram is needed, our findings facilitate oncologists' treatment making. Rigorous prospective multicenter clinical trials examining this topic are needed.

## AUTHOR CONTRIBUTIONS

**Weigang Xiu:** Investigation (equal); methodology (equal); writing – original draft (equal). **Jincheng Zheng:** Methodology (equal); software (equal). **Yuwen Zhou:** Methodology (equal); software (equal). **He Du:** Methodology (equal); validation (equal). **Jiayu Li:** Methodology (equal); validation (equal). **Wei Li:** Methodology (equal); software (equal). **Fei Zhou:** Methodology (equal). **Caicun Zhou:** Conceptualization (equal); project administration (equal). **Fengying Wu:** Conceptualization (lead); project administration (equal); writing – review and editing (equal).

## FUNDING INFORMATION

This work was supported by Clinical Research Plan of SHDC (No. SHDC2020CR4001), Shanghai Nature Science Foundation (20ZR1447100), and National Nature Science Foundation (81902314).

## CONFLICT OF INTEREST STATEMENT

The authors declare that they have no potential conflict of interest related to this research or to authorship or publication of this article.

## ETHICS STATEMENT

The Medical Research Ethics Committee of Shanghai Pulmonary Hospital Affiliated with Tongji University approved this study. Informed written consent was obtained from all study participants in accordance with the hospital ethics committee's guidelines. The study methodology was based on approved guidelines and relevant regulations.

## Data Availability

The original contributions presented in the study are included in the article/supplementary material. Further inquiries can be directed to the corresponding author.
